# Characterization of organic matter of plants from lakes by thermal analysis in a N_2_ atmosphere

**DOI:** 10.1038/srep22877

**Published:** 2016-03-08

**Authors:** Fei Guo, Fengchang Wu, Yunsong Mu, Yan Hu, Xiaoli Zhao, Wei Meng, John P. Giesy, Ying Lin

**Affiliations:** 1 Department of Chemistry, University of Science and Technology of China, Hefei, Anhui, China; 2 State Key Laboratory of Environmental Criteria and Risk Assessment, Chinese Research Academy of Environmental Sciences, Beijing 100012, China; 3 Department of Veterinary Biomedical Sciences and Toxicology Centre, University of Saskatchewan, Saskatoon, Saskatchewan, Canada; 4 Department of Zoology, and Center for Integrative Toxicology, Michigan State University, East Lansing, MI, USA; 5 Assessment Center of Environmental Engineering, Department of Environmental Protection, Beijing, 100012, China

## Abstract

Organic matter (OM) has been characterized using thermal analysis in O_2_ atmospheres, but it is not clear if OM can be characterized using slow thermal degradation in N_2_ atmospheres (STDN). This article presents a new method to estimate the behavior of OM in anaerobic environment. Seventeen different plants from Tai Lake (Ch: *Taihu*), China were heated to 600 °C at a rate of 10 °C min^−1^ in a N_2_ atmosphere and characterized by use of differential scanning calorimetry (DSC) and thermal gravimetric analysis (TGA). DSC chromatograms were compared with 9 standard compounds. Seven peaks were observed in DSC chromatograms, 2 main peaks strongly correlated with biochemical indices, and one main peak was a transitional stage. Energy absorbed by a peak at approximately 200 °C and total organic carbon were well correlated, while energy absorbed at approximately 460 °C was negatively correlated with lignin content. Presence of peaks at approximately 350 and 420 °C varied among plant biomass sources, providing potential evidence for biomass identification. Methods of STDN reported here were rapid and accurate ways to quantitatively characterize OM, which may provide useful information for understanding anaerobic behaviors of natural organic matters.

Organic matter (OM) is important as complexing agents or adsorbents of environmental pollutants found in surface waters. OM influences the forms, toxicity, and bioavailability of pollutants; however, conventional methods of characterizing OM are unsatisfactory because they do not capture the complete continuum of behavior of OM^1^. Thermal methods, which have simple pretreatment and short experimental times, have been shown to be easy, fast, relatively inexpensive yet reliable for characterizing OM^2^. To date, studies of applications of differential scanning calorimetry (DSC) and thermal gravimetric analysis (TGA) on OM have focused on thermal oxidation of soil organic matter (SOM) or plant biomass in aerobic atmospheres^3^. Thermal experiments in anaerobic atmospheres focus primarily on mechanisms and products of rapid thermal composition of biomass at stable temperature; however, few investigations of OM by use of slow thermal degradation in N_2_ atmospheres (STDN) have been conducted to date.

Behavior of soil organic matter (SOM) during slow thermal oxidation (STO) in aerobic atmospheres has been well characterized, and stages of the STO process have been qualitatively described. SOM can be divided into three categories during thermal oxidation: labile organic matter (200–380 °C), recalcitrant organic matter (380–475 °C), and refractory organic matter (475–650 °C), including black carbon^4^,^5^,^6^,^7^. Thermal analyses focus on the two former categories, corresponding to the first two peaks of the DSC curve, which are considered to be aliphatic and carboxylic groups (300 °C) and aromatic groups (450 °C), respectively^8^,^9^,^10^. Furthermore, thermal analysis of extracted humus can be used to evaluate the degree of humification^4^,^11^.

In recent years, TGA and DSC have been applied widely in studies of stability of SOM and thermal indexes have been shown to be related to land-use types, tillage practices, and even forest fires^7^,^12^,^13^,^14^,^15^,^16^,^17^,^18^. The relationship between thermal and biochemical indexes has also been found to be a potential, rapid method for inspection of SOM^7^. Moreover, thermal analysis has been demonstrated to be useful for identification of types of vegetation from which organic matters in soils and sediments have derived^10^.

However, slow thermal degradation in the N_2_ atmosphere (STDN) of OM is poorly understood. Pyrolysis is a rapid, thermal, degradation technique conducted under a N_2_ atmosphere that has been widely applied to characterization of structures of natural macromolecules. Although pyrolysis has been thoroughly investigated, few studies of STDN have investigated the process qualitatively^19^. For example, when thermal degradation characteristics of pistachio shells in an N_2_ atmosphere at different rate of heating were studied by use of a thermo-gravimetric analyzer, two main peaks were observed before and after 350 °C in the DTG curves^20^. A similar study was conducted by using DSC and TGA to monitor the composting of solid wastes such as vegetable waste and sludge^2^,^21^. Nevertheless, studies of anaerobic fermentation of biomass have usually been based on thermal oxidation data^22^,^23^,^24^,^25^, so advanced understanding of STDN of OM is still needed.

Ongoing thermal analyses of OM have revealed differences between DSC curves of thermal oxidation and STDN. The former is composed of two or three exothermic peaks, while the latter is composed of two or three endothermic peaks and one exothermic peak, and there are several indistinct endothermic peaks associated with exothermic processes^2^,^21^.

STDN has the advantages of being rapid, convenient, and quantifiable. Parameters characterizing the pyrolysis process, such as change in mass, absorbed heat (amount and rate), and released heat (amount and rate) can be determined throughout the heating period, and the results can be combined with other biochemical indexes. Thus, it is an effective method for quantitative characterization of OM and identification of their sources. However, DSC scanning is a difficult process because of the complexity of OM. Specifically, fusions, decomposition and polymerization occur as the temperature increases in reactions that could be endothermic or exothermic. Moreover, superposition can occur during each process. Accordingly, it is important to identify each peak during analysis.

The test method used for plant samples is more mature than that used for natural organic matter. Thus, in this study, 17 samples of plant materials were selected to interpret the characteristics at each stage of the STDN process based on comparison to standard materials. Specifically, this study investigated: a) whether the characteristic peak at 300 °C is a shoulder peak generated from two superposed exothermic peaks or a transition stage between an endothermic peak and an exothermic peak; b) whether the characteristic peak at 460 °C is an endothermic peak or a transition stage between two exothermic peaks; c) the relationship between peaks of OM at different stages and their components; d) the relationship between characteristic peaks of OM at different stages and other biochemical indexes.

## Results

### Differential scanning calorimetry analysis

**Figure 1:**
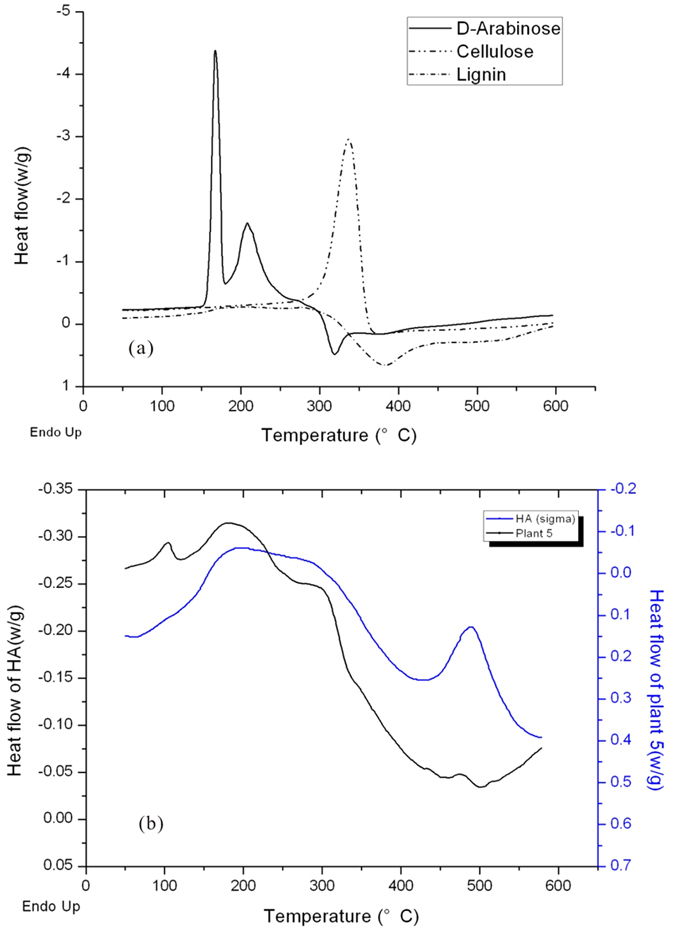
Differential scanning calorimetry (DSC) scans of D-arabinose, cellulose and lignin (a) and DSC scans of HA from Sigma and plant 5 from Taihu lake (b).

There were seven characteristic peaks observed in DSC curves of plant biomass. These peaks might have represented various C contents and different natures of the organic materials in the plant samples (Table 1). Selected DSC scans for plant biomass and standard samples are illustrated (Fig. 1), some of which showed differences in peak position, area, and height. The first endothermic peak (F1) appeared between 93° and 110 °C, while the second endothermic peak (F2) occurred at 175–217 °C. A third endothermic peak (F3) was observed at 277–311 °C in every scan of plant materials, while another endothermic peak (F3b) near F3 was observed between 344 °C and 358 °C and the fourth endothermic peak (F4) was observed at 404–437 °C in some of the scans. A remarkable exothermic peak (Fx) occurred in the range of 303–450 °C, with an endothermic peak (F5) occurring among it in the range of 444–480 °C. F2, F3, F5, and Fx were found in each scan, while F1, F4, and F4b were only found in some scans. Three peaks were found in at least one curve, while all seven peaks were found in the DSC curve produced by amaranth.

To evaluate the seven peaks observed in plant materials, nine representative organic materials were analyzed and DSC curves of standard samples were compared with sans of plant materials. Monosaccharide DSC curves (Fig. 1a) showed three endothermic peaks at 159–170 °C, 207–222 °C, and 292–312 °C, as well as an exothermic peak at 305–343 °C. These were the only two peaks observed in scans of cellulose (Fig. 1a), an endothermic peak at 336.2 °C and an exothermic peak at 378.6 °C. Scans of lignin (Fig. 1a) exhibited two endothermic peaks at 205.4 °C and 278.0 °C and an exothermic peak at 384.4 °C. In contrast to monosaccharide (monosaccharide) and cellulose, heat released by lignin decreased rapidly after the exothermic peak, manifesting as the curve nearing the x-axis. Black carbon continued to absorb heat, and the absorption rate increased at above 200 °C.

Thermal behaviors of plant samples derived from the DSC experiments were evaluated based on standard sample data, and curve of *Euryale ferox* Salisb. which exerted most characteristic peaks was shown in Fig. 1b for example. F1 peaks were greatest in the range of 93–110 °C, occurring from 76 °C to 120 °C. These peaks were not due to evaporation of water because samples had been heated to 110 °C to evolve physical-sorbed water. Moreover, this peak could not represent decomposition of organic materials because they were observed when the samples were reheated. Hence, this peak was likely caused by melting or glass transition^26^,^27^ of small molecules. F2 peaks were greatest between 175 °C and 217 °C, occurring from 130 °C to 210 °C. A melting peak, thermal degradation peak of monosaccharide, and the first degrading peak of lignin were found in this temperature range, while no remarkable thermal transition of cellulose occurred. However, the first endothermic peak of lignin and the main endothermic peak of monosaccharide occurred at almost the same temperature (205 °C). Therefore, the F2 peaks observed were likely the superposition of several thermal processes and cannot be individually characterized based on the data generated in this experiment.

F3 peaks were concentrated between 277 °C and 311 °C, occurring from 260 °C to 330 °C. F3 peaks were observed in each scan of 17 samples with considerable peak area, some of which showed as shoulder peaks (see Supplementary Fig. S1); thus, F3 peaks could be an endothermic degrading process. However, no endothermic peaks were observed in scans of standard samples in this temperature range. In this temperature range, scans of monosaccharide show the end of the first endothermic peak, while scans of cellulose were in the process of heat absorption, 30 °C before its endothermic peak. This temperature was between the end of the second endothermic peak and the exothermic peak of the scan of lignin. Therefore, more evidence was needed to confirm the thermal processes responsible for generation of the F3 peak. As shown in Table 2, peaks F3b and F4 were only observed for some samples. Peak F3b was detected in 50% of samples, and all of the scans of submerged plants. Peak F4 was detected in some floating plants and terrestrial plants, and 27% of the total samples. A decomposition peak between 443 °C and 470 °C was detected in DTG curves, showing the same characteristic with peak F4 of DSC curves among the four categories. While no weight loss peak occurred in the temperature range of peak F3b. Due to limitations imposed by the amount of sample and a lack of relative STDN data, these two peaks could not be well explained. Nevertheless, peaks F3b and F4 provide potential evidence that can be useful for identification chemicals in biomass because the scan peaks varied among types of plant biomass.

DSC curves of plant biomass showed a lengthy exothermic process above 300 °C, and the beginning and end of the exothermic peaks were not very clear. The range 280–450 °C was selected for F5 peaks according to the exothermic characteristics of the standard samples. Peak Fx represents the main exothermic process suggested by the corresponding thermal behavior of all standard samples. Peak F5 was observed in the middle of the Fx peak, primarily between 444 °C and 480 °C, but occurring from 430 °C to 600 °C; however, the peak area of F5 was far smaller than that of F2. Lignin was the only standard material showing corresponding peaks in DSC scans, while monosaccharide and cellulose remained stable in this temperature range. F5 peaks in lignin scans behaved like transitional peaks between two exothermic processes.

### Thermal gravimetric analysis

**Figure 2:**
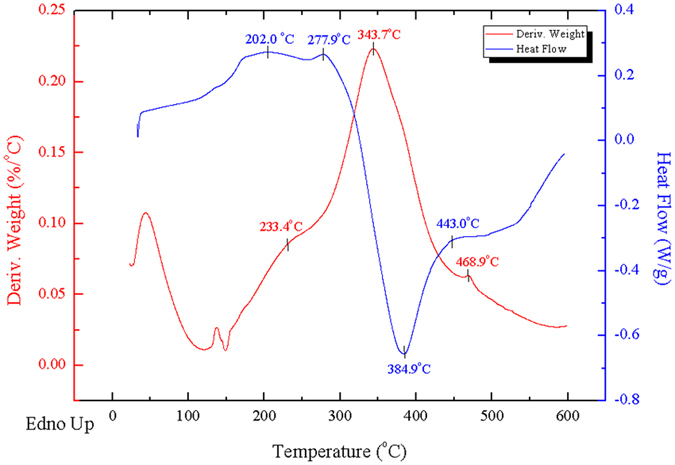
DSC and DTG overlay analysis of lignin (DSC, differential scanning calorimetry; DTG, derivative thermogravimetric analysis).

To verify if degradation occurred in DSC peaks and confirm the thermal significance of each DSC peak, DSC and DTG curves were superposed and analyzed (Fig. 2). The DSC-DTG relationship is the foundation of plant biomass behavior analysis. Monosaccharide reaches an endothermic peak at approximately 210 °C and achieves a weak loss of mass peak at 230 °C. It then exhibits an exothermic peak at approximately 330 °C, followed by a strong loss of mass peak at 340 °C, then a slow heat-releasing and slow loss of mass after 400 °C. For more stable cellulose, a strong endothermic peak was observed greater than 335 °C, with a small rate of loss of mass occurring, suggesting that the primary degradation of cellulose did not occur during the endothermic stage. Cellulose then rapidly enters an exothermic stage, peaking at 378 °C, with a loss of mass peak starting at 380 °C and ending at 450 °C. Lignin reaches the first endothermic peak at 205 °C and shows a weak loss of mass loss peak at 232 °C, while a smaller endothermic peak was observed at 277 °C followed by a strong exothermic peak. Additionally, strong loss of mass occurs at 343 °C, while exothermic processes peak later when the rate of loss of mass decreases.

**Figure 3:**
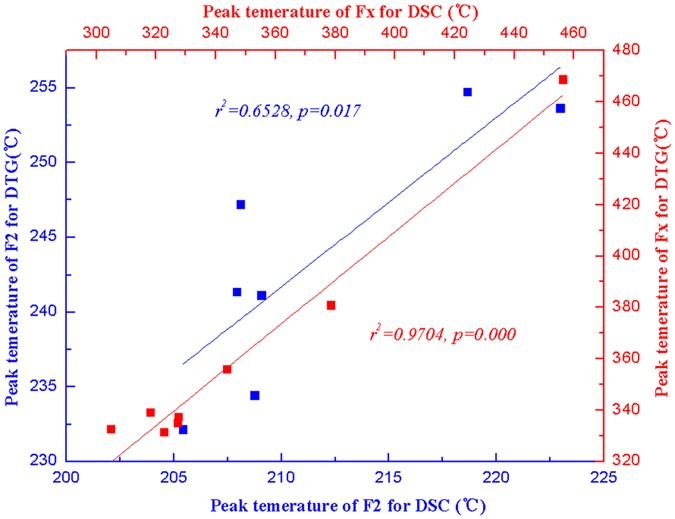
Lag relationship of peak F2 and peak Fx in DSC-DTG scans of standard samples (glucose, xylose, D-Arabinose, L-Arabinose, L-Rhamnose, D-Galactose, cellulose, lignin).

Lag rule of DSC-DTG peaks. As shown in Fig. 3, degradations lag behind thermal processes. The first degradation occurred at 210 °C, which was approximately 20 °C later than the endothermic process, while the second degradation occurred at 330 °C, approximately 10 °C later than the exothermic process.

Peaks of plant biomass were certified by use of DSC-DTG data and the standard samples. There was no obvious loss of mass peak and a lesser rate of loss of mass in the F1 peak at 93–110 °C, suggesting that peak F1 represents a melting process, which is concordant with the DSC results. Peak F2 represents the starting stage for STDN of plant biomass, corresponding to small molecules melting, endothermic processes, and preliminary degradation of monose and lignin. The rate of degradation begins to rise, although the rate was not great in this range. There was a loss of mass peak in the DTG curves 30 °C after the F2 peak (270 °C), which corresponds to the Lag rule of the DSC-DTG peak of the standard samples. Taken together, these findings suggest that peak F2 represents a recombination process of endothermic melting in the first half and endothermic degradation in the latter half.

DTG curves showed the main loss of mass peak at 10–20 °C after the F3 peak. Based on the lag rule of the DSC-DTG peak, it is likely that the F3 peak was an endothermic degradation process. However, analysis of the standard samples did not support this finding. The main degradation peak of monosaccharide at 330 °C is the result of exothermic processes at 320 °C, while the main degradation peak of cellulose and lignin correspond to the exothermic peak at 380 °C. Scans of monosaccharide revealed a transitional flat at 250–310 °C between endothermic and exothermic processes, with similar shapes to most F3 peaks. The relatively smaller peak area of F3 is further evidence that this peak does not represent an endothermic degradation process. Consequently, peak F3 represents a stable stage before the strong exothermic degradation, but not an endothermic loss of mass.

In the temperature range of peak F5, lignin was the only standard sample showing DSC and DTG peaks, and no loss of mass was observed in the monosaccharide or cellulose scans. Most samples with peak F5 showed larger loss of mass peaks at 460 °C, coinciding with the behavior of lignin.

## Discussion

**Figure 4:**
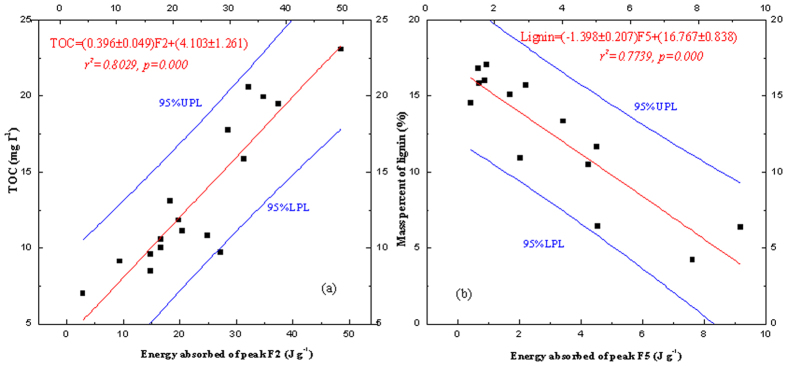
Correlation between peak F2 and TOC (a) and correlation between peak F5 and content of lignin (b) (DSC, differential scanning calorimetry; TOC, total organic carbon).

Biochemical and heating indices of plant biomass were quantitatively analyzed to verify the physical significance of each peak. The sample names, total organic carbon (TOC) and components of 17 samples can be found as Supplementary Table S1. There was a positive and approximately linear correlation between the peak F2 area (energy absorbed per gram of plant sample), and total organic carbon, and the correlation coefficient (*r*) was 0.903 (*p* = 0.000) (Fig. 4a).

Peak areas of F2 and TOC were well correlated; however, the correlation coefficient (*r*) of the peak area (with *p*) of F2 and extract, cellulose, hemicelluloses, and lignin were only 0.529 (0.029), 0.297 (0.246), 0.611 (0.009), and 0.791 (0.000), respectively. These findings indicate that peak F2 is a combination of different organic materials. The peaks in sample 1, 4, and 10 were close to the monosaccharide melting peak, while those in samples 2, 8, 12, and 13 were similar to the lignin endothermic peak, and the remaining peaks fell between these two values. However, peak F2 never appeared to be related to any composition. For example, in the DSC curve of *Alternanthera philoxeroides* (Mart.) Griseb., the F2 peak occurs at 177.4 °C, while the heat absorption rate peaks at 0.045 W/g. Conversely, the rate of thermal decomposition at 177.4 °C (0.0382%/°C) was much slower than the nearest peak at 270.49 °C (0.2792%/°C). Hence, a strong endothermic process occurs, but with no significant mass loss owing to the breakage of hydrogen bonds or melting of small molecule organic matter. Overall, the processes resulting in the F2 peak are mainly dominated by absorbing energy, as well as minor decomposition.

The absorbed heat of peak F3 was poorly correlated with each component and total organic matter, suggesting that peak F3 was not an endothermic decomposing peak, but instead represents a smooth transition period. This is consistent with the finding that peak F3 was a baseline transition based on comparison with the standard sample, the lag rule of the DSC-DTG peak, and component correlation analysis, although some evidence suggests that peak F3 may be an endothermic pyrolysis peak. Similar transition phenomenon was observed in DSC experiment of sewage sludge^21^.

The area of peak F5 exhibited a negative correlation with lignin content (Fig. 4b), and the correlation coefficient (r) was 0.890 (*p* = 0.000). One possible explanation for this observation is that lignin form biochar at high temperature via an exothermic process, and the gap between the two exothermic processes presents as an endothermic peak. Thus, peak F5 represents the gap between endothermic processes instead of an endothermic pyrolysis peak. Nevertheless, this peak can be used to characterize lignin content.

HA scan curve (Fig. 1b) exhibited peaks F2, F5, and Fx. Peaks F2 and Fx were smaller, while peak F5 was larger than those of *Euryale ferox* Salisb. scan curve (Fig. 1b).HA can be characterized based on knowledge from plant sample scans, meaning most labile carbon degraded, while stable carbon became the component of HA.

Based on all experimental findings, STDN of plant biomass could be divided into three stages. Peak F2 represents endothermic, primary degradation processes that occur at a relatively slow rate. This was followed by peak F3, reflecting a transitional stage in which energy was still being absorbed, while the Fx peak represents severe exothermic activity and rapid loss of mass. This study provides a foundation for understanding the biomass STDN. Results revealed that several thermal behaviors overlap, making it difficult to identify components in OM. However, thermal indexes revealed significant quantitative relationships with biochemical indexes, and it is anticipated that slow thermal degradation in an N_2_ atmosphere could be a potential method for characterization and even identification of OM.

## Methods

### Preparation of Samples

Seventeen samples of plant materials collected from Tai Lake (Ch: *Taihu*) were used in this study. Samples were classified into four categories (emergent plants, submerged plants, floating plants, and terrestrial plants). The materials collected were dried at 90 °C for 1–2 h and then completely dried at 60 °C for 12–24 h. After drying, they were ground to pass through a 1-mm sieve. Standard samples were analyzed using the same experimental protocol to enable comparison of the seven DSC peaks from the plant samples. Eight representative organic materials, glucose, xylose, D-arabinose, L-arabinose, L-rhamnose, D-galactose, cellulose, and lignin were selected and black carbon was included to investigate its probable influence. Humic acid (HA) (Sigma-Aldrich, St. Louis, USA) was scanned to verify results derived from plant sample scans.

#### Thermal gravimetric analysis

Thermal gravimetric analysis was performed by use of a TGA Q50 thermo-gravimetric analyzer (TA Instruments, USA) to evaluate the thermal stability of samples and evolution of physic-sorbed water. In this study, TGA was used to scan approximately 5 mg of samples from ambient (approximately 30 °C) to 800 °C at 10 °C min^−1^ under nitrogen gas (99.99% purity) flowing at 80 mL min^−1^.

### Differential scanning calorimetry

Differential scanning calorimetry was performed by use of a Model DSC Q20 Differential Scanning Calorimeter (TA Instruments). Briefly, 5–10 mg of powdered sample was placed into a standard aluminum pan (Lot no. 900786.901; TA Instruments). To improve accuracy of the DSC, the mass of the sample pan and reference were both weighed to ensure that the difference was less than 0.1 mg. Samples were then placed under an atmosphere of nitrogen gas (99.99% purity) at a flow rate of 100 mL min^−1^ and each sample pan was conditioned to remove the physic-sorbed water by heating from ambient to 110 °C at 10 °C min^−1^, holding for 30 min, and then cooling to ambient. DSC was then conducted while heating from ambient to 600 °C at 10 °C min^−1^.

TOC was measured by use of a total organic carbon analyzer (TOC-VCPH; Shimadzu, Japan) by the non-dispersible infrared absorption (NDIR) method. Cellulose, hemicellulose, lignin, and abstract matter were determined according to the Determination of Structural Carbohydrates and Lignin in Biomass method recommended by the National Renewable Energy Laboratory (see Supplementary Methodology). Universal Analysis 2000 software was used to determinate thermal indexes such as peak area and peak temperature of each peak.

## Additional Information

**How to cite this article**: Guo, F. *et al*. Characterization of organic matter of plants from lakes by thermal analysis in a N_2_ atmosphere. *Sci. Rep.* **6**, 22877; doi: 10.1038/srep22877 (2016).

## Supplementary Material

Supplementary Information

## Figures and Tables

**Table 1:** Thermal indices of plant samples (Fn is an endothermic or exothermic peak, and An is area of Fn, which means energy absorbed.

No.	F1 (J/g)	T1 (°C)	F2 (J/g)	T2 (°C)	F3 (J/g)	T3 (°C)	F4 (J/g)	T4 (°C)	F4 b(J/g)	T4 b(°C)	F5 (J/g)	T5 (°C)	Fx (J/g)	Tx (°C)
1	1.37	104.25	14.85	177.38	8.42	308.82							−31.81	313.35
2	5.90	107.46	27.23	206.66	9.93	301.65	1.20	358.07			4.51	469.31	−60.85	303.26
3	0.72	98.73	18.27	193.13	12.62	309.28							−75.10	312.94
4	0.03	99.15	24.92	175.87	37.92	318.98					0.88	471.13	−37.31	333.43
5	4.03	104.89	34.87	180.87	30.94	303.86			0.41	437.09	2.04	474.84	−30.29	320.30
6	2.55	95.04	31.36	198.72	25.35	303.80					0.44	473.27	−29.40	319.79
7	2.22	93.52	19.82	193.87	15.20	289.89					3.44	461.09	−53.35	300.87
8	6.72	110.14	2.89	217.72	15.06	311.77			1.47	428.33	0.71	474.32	−16.08	329.59
9			32.11	195.67	16.46	292.18	4.76	344.93			4.26	444.70	−71.03	300.03
10	3.42	100.61	14.87	177.27	11.23	309.21	2.95	353.99	1.15	423.77	2.23	472.88	−13.38	321.91
11	3.38	107.98	9.35	201.77	14.61	307.22	2.84	351.27					−9.669	321.90
12			16.58	209.06	17.24	297.29	1.26	347.00			0.69	476.09	−37.88	308.69
13			20.40	213.97	10.95	287.71	6.74	349.08			1.71	445.94	−39.51	295.86
14			16.69	184.03	17.28	285.62	2.52	347.31			0.96	451.43	−29.63	301.57
15	1.97	93.05	48.58	187.50	19.39	277.75	8.58	346.72	10.94	436.40	7.62	466.68	−136.3	280.32
16			37.43	202.06	9.41	314.38			0.53	404.17	4.56	480.47	−101.1	316.29
17			28.58	186.50							9.19	453.97	−129.4	323.12

Tn is the peak temperature of Fn (n is 1, 2, 3, 4, 4b, 5 and x respectively). Blank means no peak was observed in this scan).

**Table 2:** Probability of F3b and F4 peaks shown in different plant types.

Peak type	Emerged plants	Submerged plants	Floating plants	Terrestrial plants	All plants
F3b	25%	33%	100%	50%	50%
F4	0	50%	0	50%	27.5%
