# Supplementary material for: Characterization of organic matter of plants from lakes by thermal analysis in a N2 atmosphere
Source: Sci Rep. 2016 Mar 8;6:22877. doi: 10.1038/srep22877 (PMC4782168; doi:10.1038/srep22877)
Supplement: Supplementary Information [file srep22877-s1.pdf]

Supplementary Information

Submitted to: *Nature: Scientific Reports*

Characterization of organic matter of plants from lakes by thermal analysis in a N_2 atmosphere

Fei Guo, Fengchang Wu[*], *Yunsong Mu, Yan Hu, Xiaoli Zhao, Wei Meng, John P. Giesy, Ying Lin*

Supplementary Table S1. TOC and components of plant samples (TOC, total organic carbon; Percentages are calculated based on dry mass).

NO.	Sample name	Category	TOC	Extract	Cellulose	Hemicellulose	Lignin
					mass %		
**1**	*Alternanthera philoxeroides* (Mart.) Griseb.	Emerged plants	9.56	7.90	30.43	12.83	15.81
**2**	*Polygonum* L.	Emerged plants	9.7	13.12	32.97	20.20	11.66
**3**	*Zizania latifolia* (Griseb.) Stapf	Emerged plants	13.07	15.13	31.39	20.10	11.45
**4**	*Phragmites communis* Trin.	Emerged plants	10.79	7.70	33.83	22.88	16.00
**5**	*Euryale ferox* Salisb.	Floating plants	19.93	15.32	30.64	19.94	10.92
**6**	*Nelumbo nucifera* Gaertn.	Floating plants	15.86	16.38	27.35	20.49	14.53
**7**	*Hydrocharis dubia* (Bl.) Backer	Floating plants	11.82	10.87	29.34	16.43	13.33
**8**	*Lemna minor* L.	Floating plants	7	10.85	28.87	16.23	15.79
**9**	*Nymphoides peltatum* (Gmel.) O. Kuntze	Floating plants	20.55	15.54	30.57	19.42	10.47
**10**	*Trapa bicornis* Osbeck	Floating plants	8.49	9.27	29.27	18.34	15.69
**11**	*Myriophyllum spicatum* L.	Submerged plant	9.11	6.20	28.10	15.49	15.15
**12**	*Potamogeton distinctus* A.Benn.	Submerged plant	10	7.17	30.93	16.47	16.79
**13**	*Sonchus oleraceus L.*	Submerged plant	11.13	7.92	26.82	16.14	15.06
**14**	*Hydrilla verticillata* (L. f.) Royle	Submerged plant	10.55	8.30	26.70	20.50	17.07
**15**	*Amaranthus tricolor* L.	Terrestrial plant	23.06	12.20	28.42	20.50	4.22
**16**	*Aeschynomene indica* L.	Terrestrial plant	19.46	10.60	43.05	20.43	6.40
**17**	*Chenopodium glaucum* L.	Terrestrial plant	17.73	13.68	27.02	21.13	6.35

Supplementary Figure S1. Peak F3 of plant 2, 5, 8, and 15

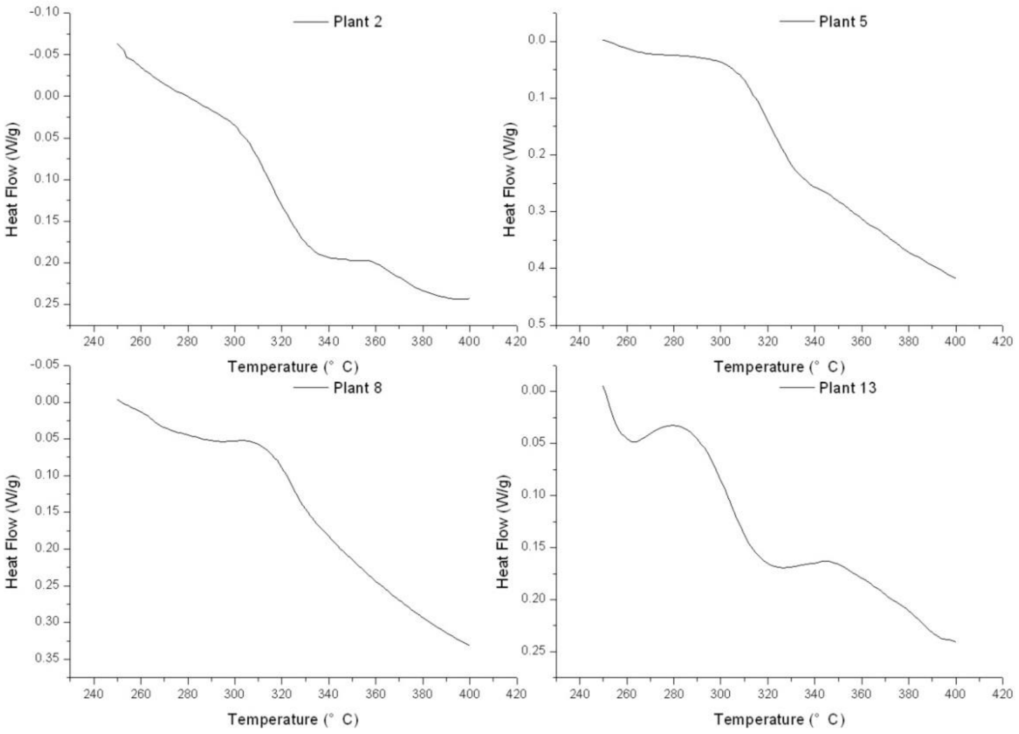

Supplementary Methodology

Methodology of the Determination of Structural Carbohydrates and Lignin

1. Weigh 300.0 ±10.0 mg of the sample into a tared pressure tube. Each sample should be analyzed in duplicate.
2. Add 3.00 ±0.01 mL of 72% sulfuric acid to each pressure tube. Place the pressure tube in a water bath set at 30 ±3 ℃ and incubate the sample for 60 ±5 minutes.
3. Dilute the acid to a 4% concentration by adding 84.00 ±0.04 mL deionized water using an automatic burette.
4. Place the tubes in an autoclave safe rack, and place the rack in the autoclave. Autoclave the sealed samples and sugar recovery standards for one hour at 121 ℃.
5. After completion of the autoclave cycle, allow the hydrolyzates to slowly cool to near room

temperature before removing the caps.

Analyze the sample for acid insoluble lignin as follows

6. Transfer an aliquot, approximately 50 mL, into a sample storage bottle. This sample will be used to determine acid soluble lignin as well as carbohydrates.
7. Use deionized water to quantatively transfer all remaining solids out of the pressure tube into the filtering crucible.
8. Dry the crucible and acid insoluble residue at 105 + 3 ℃ until a constant weight is achieved. Remove the samples from the oven and cool in a desiccator.
9. Place the crucibles and residue in the muffle furnace at 575 ±25 ℃ for 24 ±6 hours. Carefully remove the crucible from the furnace directly into a desiccator and cool for a specific amount of time. Weigh the crucibles and ash to the nearest 0.1 mg and record the weight.

Analyze the sample for acid soluble lignin as follows

10. Using the hydrolysis liquor aliquot obtained in step 6, measure the absorbance of the sample at an appropriate wavelength on a UV-Visible spectrophotometer.

Analyze the sample for structural carbohydrates

11. Analyze the calibration standards, and samples by HPLC using a Shodex sugar SP0810 or Biorad Aminex HPX-87P column equipped with the appropriate guard column.
